# Beliefs about medication as predictors of medication adherence in a prospective cohort study among persons with multiple sclerosis

**DOI:** 10.1186/s12883-021-02149-0

**Published:** 2021-03-25

**Authors:** Efrat Neter, Lea Glass-Marmor, Anat Wolkowitz, Idit Lavi, Ariel Miller

**Affiliations:** 1 Ruppin Academic Center, 3 Bait, Ruppin Academic Center, 4025000 Emeq Hefer, Israel; 2 Rappaport Faculty of Medicine, Technion Institute of Technology, Haifa, Israel; 3 Department of Community Medicine & Epidemiology, Carmel Medical Center, Haifa, Israel; 4 Multiple Sclerosis Center & Department of Neurology, Carmel Medical Center, Haifa, Israel

**Keywords:** Disease modifying therapy, Medication adherence, Medication beliefs, multiple sclerosis, participatory medicine, Patient reported outcomes, Persistence

## Abstract

**Background:**

Though adherence to disease-modifying therapies (DMTs) among persons with multiple sclerosis (PwMS) varies and is often below 80%, only few prospective studies on adherence examined predictors beyond demographic and clinical characteristics.

**Objectives:**

Identify antecedents to adherence and persistence to DMT in a prospective design among PwMS.

**Methods:**

PwMS (*n* = 186) were prospectively assessed at three time points: baseline, 6 (Time 1) and 12 months later (Time 2). Clinical, demographic information and patient-reported medication beliefs, illness perceptions, medication habits, perceived health and affect were surveyed in-person. Adherence and persistence were assessed by a combination of self-reports and retrospective review of medication claims.

**Findings:**

PwMS were 69.9% (Time 1) and 71% (Time 2) adherent to their DMTs and 64.5.9% were persistent. Beliefs about Medications were consistently predictive at both time points (baseline to Time 1 and Time 1 to Time 2) of medication adherence and persistence whereas other perceptions were predictive in some analyses; clinical and demographic characteristics were mostly not predictive of adherence nor persistence. The prospective association of beliefs about medication with adherence held also in multivariate analyses (OR = 0.88, 95% CI 0.78–0.99, *p* = 0.029).

**Conclusions:**

Adherence and persistence are predicted by medication beliefs of PwMS. As medication beliefs are modifiable, they should be assessed periodically and targeted as a focus of tailored interventions aimed to improve adherence and consequently health outcomes in PwMS.

**Registration:**

Clinical trials registry #NCT02488343, date: 06/08/2015.

## Background

There is widespread recognition that adherence to medication is key to successful health care of persons with Multiple Sclerosis (PwMS) [1–5] yet reviews on disease-modifying therapies (DMT) medication-taking among PwMS estimate adherence as ranging between 41 and 88% [1] and persistence ranging from 16 to 27% [2]. Adherence is especially challenging to PwMS taking DMTs. Despite of the long-term nature of the chronic condition that requires medication taking for long periods, the need for DMTs may be less obvious during periods of disease inactivity which may turn PwMS complacent on adherence. Concomitantly, adverse side effects of DMTs reduce quality of life of PwMS [6], and are often associated with decreased adherence.

Medication nonadherence is widely recognized as a common and costly problem [7], as nonadherence reduces the patient’s potential benefits from treatment [8] and increases healthcare costs [9]. Medication-taking behavior has two main aspects: adherence and persistence [10]. Adherence refers to the extent of correspondence between medication-taking behavior and the recommendations made by the provider with respect to the timing, dosage, and frequency whereas persistence refers to staying on (same) treatment [11, 12].

The World Health Organization adherence model posits that adherence is determined by the interplay of five sets of factors: social and economic (e.g., age, ethnicity, education), health care system (e.g., type of insurance), condition-related (e.g., duration, comorbidity), therapy-related (e.g., type of medication, complexity of regimen, side effects) and patient-related [7]. Factors most often studied are social-economic and patient-related, as the first is easier to measure and the latter is considered potentially modifiable in interventions, including perceptions on illness, medication beliefs, habits in medication and affective states [13, 14]. The latter are assessed mostly by patient-reported outcomes, increasingly used in MS [15]. Most studies on adherence among PwMS examined the social-economic factor, and only few studies investigated patient-related factors [16–18] or therapy-related factors [2, 19–21].

Considerable variation is evident in the measurement of adherence, with no single gold standard [9, 22]. Hence, different measures (e.g., patients’ reports, medication possession ration (MPR), and electronic monitoring devices (EMD)) are regarded as measuring different phenomena, each with shared and unique variability related to clinical outcomes [22, 23]. Most studies, though, rely on a single method to evaluate adherence [24, 25].

**Fig. 1:**
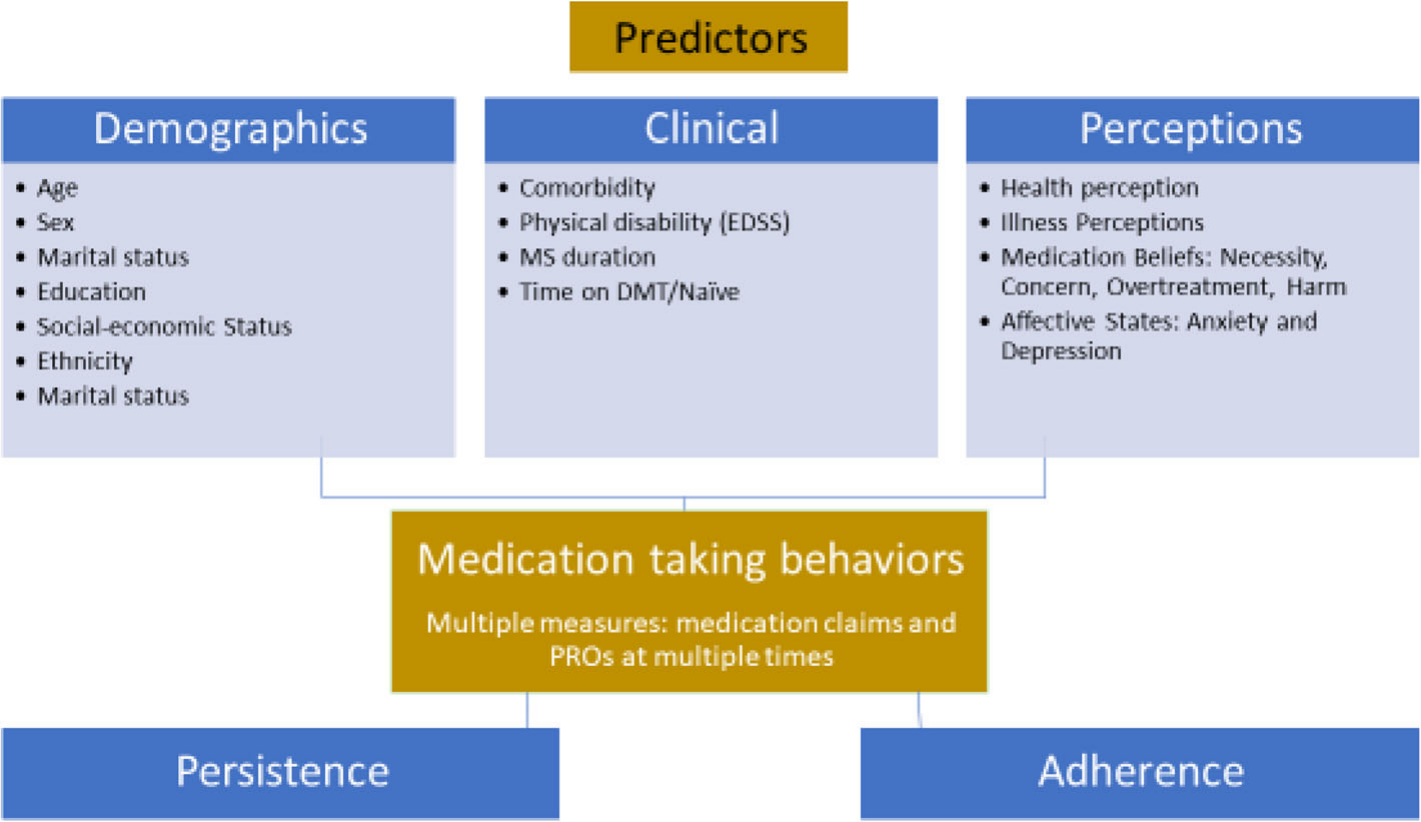
Conceptual adherence model

Only few studies in MS [18, 26–28] have used multiple measures of adherence and longitudinally examined their association with varied potential antecedents. Thus, the present study aimed at assessing both adherence and persistence using multiple measures (patient reported outcomes and medication claims) and examined their association with diverse predictors among MS patients (Fig. 1).

## Methods

### Participants and procedure

**Fig. 2:**
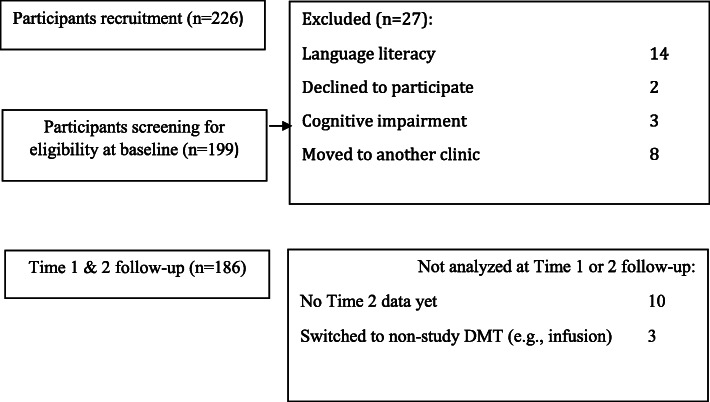
Enrollment of Participants

Persons with relapsing-remitting multiple sclerosis (RRMS) treated with DMTs at Carmel Medical Center’s specialized MS clinic in Haifa, Israel: 186 at baseline, 6 months later (Time 1) and 12 months (Time 2) since baseline. Recruitment is depicted in Fig. 2.

A prospective observational study design was used. Data were collected in a large single-center between February 2016 and February 2019 within an in-service context and is still on-going. Inclusion criteria were: RRMS diagnosis, and being at baseline on DMT of Fingolimod, Dimethyl Fumarate, Interferon beta-1a and Glatiramer Acetate. These DMTs were the most often self-administered medication prescribed at the clinic at the time. Exclusion criteria at recruitment were: language literacy, cognitive impairment, disinclination to participate and moving to another clinic. The surveys were administered prospectively at the clinic at baseline, 6 months (Time 1, median length of 6.9 months) and 12 months later (Time 2, median length of 6.8 months from Time 1) using a tablet. Neurological evaluations were made during respective clinic visits. Medication possession data were retrieved retrospectively for the same periods.

The study was approved by an Internal Review Board of Carmel Medical Center (#0061–14-CMC). All participants signed written informed consent forms confirming that they were free to leave the study at any time.

### Measures

#### Adherence and persistence

Medication withdrawal records were retrieved from the dataset of ‘Clalit Health Services’; records were available for 136 PwRRMS in the prospective study who are members of this Health Maintenance Organization (HMO) but not for 50 PwRRMS treated at the clinic who are members of other HMOs. Based on medication withdrawal records, Medication Possession Ratio (MPR) was computed for each PwRRMS based on their medication type and the initial prescription. MPR was estimated as the total days with index medication supply within the refill interval (six months between baseline and Time 1, and six months between Time 1 and Time 2), divided by the number of days between the first prescription date and the last prescription date. Using the commonly accepted threshold of MPR ≥ 80% [12], PwRRMS were categorized as adherent if they were above the threshold and non-adherent when they were below this threshold.

Patient-reported outcomes measures included Multiple Sclerosis Treatment Adherence Questionnaire (MS-TAQ) [29] and Probabilistic Medication Adherence Scale (ProMAS) [30]. The items from MS-TAQ used in this analysis evaluated whether PwRRMS did not take a prescribed dose in the last four weeks and the reported number of these doses. When non-adherence was reported, the percentage was calculated per regimen. The ProMAS is an 18-item questionnaire assessing adherence behaviors (e.g., “I have never changed my medicine use myself”, “When I am away from home, I occasionally do not take my medicines”) to which respondents indicate either ‘yes, true’ (coded 1) or ‘no, not true’ (coded 0). Higher respondent’s adherence scores represent better adherence rates. Adherence categories are low (sum score 0–4), medium-low (sum score 5–9), medium-high (sum score 10–14) and high (sum score 15–18). Internal reliabilities of the ProMAS were baseline = 0.83, Time 1 = 0.82 and Time 2 = 0.83.

An adherence score was constructed so that good adherence was defined as *either* = > 80% medication claims per regimen (medication possession ratio (MPR)), or = > 80% self-reported medication use by MS-TAQ, or being at the medium-high and high categories of ProMAS. Full details are described in a methodological report [27]. Low adherence was defined as the complement to good adherence. Persistence was defined as staying with the same medication from baseline to Time 2.

#### Predictors

*Self-Report Habit Index* (SRHI) [31] is a 12-item patient-reported outcome (PRO) assessing habit strength, specifically repetition, lack of awareness and automaticity of medication-taking behavior (in either administration route). The items were measured on a seven-point scale, ranging from ‘I completely agree’ [7] to ‘I completely disagree’ [1]. An overall score for habit strength was constructed whereby higher values denote more habit. PwRRMS reported on their medication habits at the three time points. Cronbach’s internal reliabilities were α = 0.86 and α = 0.88 for baseline and Time 1, respectively.

*Belief about Medicine Questionnaire* (BMQ) [32] was used to assess the cognitive represetations of medicines. The 18-item scale contains two five-item subscales measuring Necessity and Concerns about medication and two four-item subscales measuring Harm and Overuse. Scores on this measure were constructed so that higher scores indicate stronger beliefs in the concepts represented by the scale. Internal reliabilities were α = 0.81 for both baseline and Time 1; internal reliabilities of the subscales ranged from α = 0.71 to α = 0.83.

*Illness perceptions* were assessed by the Brief Illness Perception Questionnaire [33]. The B-IPQ includes eight items graded on a linear 0–10 response scale assessing cognitive and emotional representations of illness. Each item refers to one dimension of illness perception (consequences, timeline, identity, personal control, treatment control and coherence, and the (two-item) dimension of emotional representation). The scale was scored so that higher scores represent more negative illness perceptions. Cronbach’s internal reliabilities were α = 0.71, 0.76 at baseline and Time 1, respectively.

*Emotional states* were assessed by the Hospital Anxiety and Depression Scale (HADS) [34] which is a self-report 14-items depression and anxiety questionnaire widely used in medical settings and has been used in the past among PwMS [35, 36]. Respondents rate the degree to which they have been experienced depression and anxiety over the last week. Reliabilities were α = 0.84 and α = 0.85 at baseline for depression and anxiety, respectively.

Demographic and clinical variables examined for this study included age, gender, marital status, educational attainment and subjective social economic status, ethnicity, comorbidity, MS duration, time on current DMT and type of DMT. *Physical disability* was assessed by a neurologist using the Kurtzke Expanded Disability Status Scale (EDSS) of disease progression and neurological impairment [37].

### Statistical analysis

Descriptive analyses for demographic and clinical characteristics were conducted and reported for all participants. For categorical variables, counts and percentages are provided whereas means and standard deviations (SDs) are presented for continuous variables. Adherence was constructed so that non-adherence was defined as either detected/reported by one of the PRO or MPR [27]; it is presented across Time 1 and Time 2, and also by DMT administration route. Persistence is reported as staying with the same medication between baseline and Time 2, and reasons for discontinuation are described.

Then, adherent and non-adherent PwMS were compared in their demographic and clinical characteristics as well as their perceptions. Categorical variables were analyzed using a chi-square test, and continuous variables were analyzed using the t-test or Mann-Whitney U test (depending on the normality of distribution, tested using the Kolmogorov-Smirnov test). Statistical significance was set for *p* < 0.05. The relative contribution of variables found to be significantly different among the two groups were further evaluated using binary logistic regression analysis while adjusting also for age and gender. A similar analysis of demographic, clinical characteristics and perceptions, comparing those who persisted with their medication to those who did not persist, was conducted. No imputations were carried out on missing data.

## Results

### Patient characteristics

The study cohort consisted of 186 PwMS meeting the inclusion criteria and having follow-up data. Their demographic and clinical characteristics at baseline are depicted in Table 1. PwMS were predominantly married women. The majority had attainted post-secondary or tertiary education and assessed their economic status as average or above. Comorbidity was reported by 20.4% of PwMS and their average physical disability (as measured by EDSS) was relatively low to moderate (Mean = 2.62, SD = 2.0, Median = 2.00, IQR = 1.00–4.00). Respondents have had MS for a mean duration of 7.48 years and were taking the medication under study for a mean duration of 27.6 months.

**Table 1:** Baseline demographic and clinical characteristics (*N* = 186)

	N (%)	M (SD)
Age		40.6 (13.8)
Gender, N (%)		
Male	52 (28.0)	
Female	134 (72.0)	
Marital Status, Married	112 (60.2)	
Education*		
Secondary	57 (30.6)	
Post-secondary	30 (16.1)	
Tertiary	97 (52.2)	
Social Economic Status*		
Low	15 (8.1)	
Average and above	170 (91.9)	
Ethnicity		
Jewish	128 (69.6%)	
Arab	51 (27.7)	
Other	5 (2.7%)	
Comorbidity		
Yes	38 (20.4)	
No	148 (79.6)	
Physical disability		
EDSS at baseline		2.62 (2.00)
MS duration in years, Mean (SD)		7.48 (7.10)
Time on DMT in months, Mean (SD)		27.6 (54.6)

*Note*: EDSS: Expanded Disability Status Scale

* missing data: education on 2 cases and social economic status on 1 case

### Adherence, persistence and their prediction

Adherence scores are presented for Time 1 (69.9%) and Time 2 (71.0%) both across medication types and by administration route (injectable, oral) (see Tables 2 and 3). Adherence ranged between 66.3 and 73.8%. Persistence was at lower levels (64.5%), and reasons for discontinuation were: clinical and/or MRI deterioration (*n* = 15), pregnancy planning (*n* = 14), laboratory abnormal results (*n* = 7), and patient-reported non-tolerability (*n* = 30).

**Table 2:** Adherence and persistence to medication by time; n (%)

	Time 1	Time 2
Adherence score		
Yes^a^	130 (69.9)	132 (71.0)
No	56 (30.1)	54 (29.0)
Persisted in medication		
Yes		120 (64.5)
No		66 (35.5)

**Table 3:** Adherence (> 80%) to medication by time and route of administration^a^; n (%)

	Time 1	Time 2
Adherence score: Injectable DMTs		
Yes	53 (66.3)	52 (71.2)
No	27 (33.8)	21 (28.8)
Adherence score: Oral DMTs		
Yes	76 (73.8)	79 (72.5)
NO	27 (26.2)	30 (27.5)

*Note*: DMTs: disease modifying treatments

Adherent persons at Time 1 and Time 2 were compared to non-adherent patients on demographic (baseline), clinical characteristics and perceptual characteristics (baseline characteristics to Time 1 adherence, and Time 1 characteristics to Time 2 adherence). There was one statistically significant difference between adherent and non-adherent PwMS based on demographic and clinical characteristics, and it was not consistent across time. Specifically, adherence at Time 1 was more frequent among PwMS who had a higher social economic status (than lower social economic status), though not at Time 2.

Consistent statistically significant differences between the two groups were uncovered in perceptual characteristics (see Table 4). Specifically, adherent PwMS, compared to non-adherents, believed their medication to be less overtreatment and less harmful, both at Time 1 and Time 2. Self-rated health**,** illness perception (general score and components), habits and affective states (depression and anxiety) at baseline and Time 1 did not differ significantly between the adherence groups at Time 1 and Time 2, respectively.

**Table 4:** Patient’s Perceptions predicting adherence score at Time 1 and Time2; M (SD)

Predictors	Adherent	Non-adherent	*p*-value
Adherence score at Time 1 (predicted by baseline)			
Self-rated health	3.20 (1.22)	2.98 (1.24)	0.287
Illness Perception (score)	40.23 (12.97)	42.09 (14.17)	0.389
Medication Beliefs			
Necessity	18.34 (3.70)	16.94 (4.63)	0.083
Concern	14.38 (4.96)	15.37 (4.47)	0.219
Overtreatment	10.17 (3.37)	11.39 (3.82)	0.042
Harm	8.77 (2.92)	9.96 (3.08)	0.022
Habits	41.04 (18.23)	45.76 (17.61)	0.154
Depression	6.30 (4.92)	6.39 (4.03)	0.316
Anxiety	7.41 (5.03)	7.91 (4.30)	0.666
Adherence score at Time 2 (predicted by perceptions at Time 1)			
Self-rated health	3.21 (1.20)	2.92 (1.28)	0.163
Illness Perception (score)	40.34 (13.89)	43.74 (13.18)	0.163
Medication Beliefs			
Necessity	17.91 (3.87)	17.12 (4.43)	0.244
Concern	13.81 (5.18)	15.22 (4.88)	0.101
Overtreatment	10.07 (3.66)	12.26 (3.47)	0.000
Harm	9.00 (3.30)	10.78 (3.01)	0.001
Habits	37.83 (18.95)	37.57 (16.66)	0.932
Depression	8.28 (4.63)	9.59 (4.13)	0.097
Anxiety	8.83 (4.33)	9.67 (4.36)	0.166

Beliefs about medication were then tested as a predictor of adherence in a multivariate analysis, controlling for traditional demographic variables of age and gender. Table 5 presents the results of bivariate and multiple logistical regressions. Overtreatment and harm at baseline still predicted adherence at Time 1 in a multivariate analysis which also controlled for age, gender and social economic status (found associated in the univariate analysis). Likewise, perceptions of overtreatment and harm at Time 1 still predicted adherence at Time 2.

**Table 5:** Multiple Logistical Regression Predicting Adherence (n = 186) ^ab^

	Bivariate analyses		Multivariate analyses	
Predictors	Odds ratio (95% CI)	p-value	Odds ratio (95% CI)	*p*-value
Predicting Time 1 Adherence				
Medication Beliefs^a^				
Over-treatment	0.90 (.82–0.99)	0.045	0.90 (0.81–0.99)	0.031
Harm	0.87 (.78–0.98)	0.022	0.86 (0.77–0.97)	0.015
Predicting Time 2 adherence				
Medication Beliefs^a^				
Over-treatment	0.84 (0.77–0.93)	0.001	0.88 (.78–0.99)	0.029
Harm	0.84 (0.76–0.94)	0.002	0.89 (.78–1.01)	0.074
Predicting Time 2 Persistence				
Medication Beliefs^a^				
Concerns	1.08 (1.01–1.15)	0.030	1.09 (1.02–1.17)	0.017

*Note*: ^a^ Medication beliefs at baseline predicting adherence Time 1 and persistence; medication beliefs at Time 1 predicting adherence Time 2

^b^ Adjusted for age and gender

Persistence, just as adherence, was not predicted by demographic and clinical characteristics. Persistence, measured at Time 2, was predicted by perceptions at baseline, specifically concerns about medication and anxiety (see Table 5). A multivariate analysis which also controlled for age, gender and included anxiety and concerns about medication, resulted in male gender being the only predictive variable (OR = 2.34, 95% CI 1.07–5.14, *p* = 0.034). As concerns about medication and anxiety were highly correlated (*r* = 0.52, *p* < 0.001), the regression was also run controlling only for age and gender, and concerns about medication, age (younger) and male gender were significantly predictive of persistence (see Table 6).

**Table 6:** Treatment Persistence (Time 2) predicted by perceptual variables at baseline ^a^

Predictors	Persisted	Not persisted	*p*-value
Self-rated Health	2.87 (1.18)	2.88 (1.33)	0.939
Illness Perception	39.60 (13.07)	42.95 (13.64)	0.105
Habits	40.75 (18. 46)	46.81 (16.71)	0.074
Medication Beliefs			
Necessity	18.07 (3.97)	17.67 (4.17)	0.967
Concerns	14.05 (4.97)	15.72 (4.41)	0.028
Over treatment	10.60 (3.57)	10.41 (3.50)	0.647
Harm	9.05 (3.02)	9.23 (3.01)	0.810
Anxiety	6.82 (4.77)	8.85 (4.64)	0.006
Depression	6.16 (4.52)	6.54 (4.91)	0.754

^a^ Adjusted for age and gender

## Discussion

Adherence to DMTs in this sample of PwMS, assessed by the combination of measures, medication claims and patients’ reports [27], fell within the range reported in previous studies on medication adherence [2, 24]. The novel finding of the present study is that adherence and persistence were consistently associated prospectively with patient-related factors, specifically perceptions of medication – beliefs on the harm medication cause, their overuse and general concern. Other patient-reported perceptions (i.e., anxiety) were prospectively associated with adherence or persistence at one of the measurement time points. Adherence and persistence were largely not predicted by demographic nor MS clinical characteristics. Interestingly, the habit of medication-taking increased from Time 1 to Time 2 among most PwMS, even those less adherent.

Most (64.5%) PwMS in our sample persisted in the medication they were taking at the follow-up period. Two findings on persistence are noteworthy. First, almost half of all non-persisters (*n* = 66) stopped taking the medication following reported complaints on non-tolerability (*n* = 30). Though the decision to discontinue the medication is shared by the physician and the patient, the move is driven by patients’ perceptions, highlighting the importance of patients’ perceptions. Secondly, though both adherence and persistence were predicted by beliefs about medication, the specific beliefs differed: harm and overtreatment were prospectively associated with adherence, whereas concerns were prospectively associated with persistence.

The lack of association between demographic characteristics and adherence is different from some previous studies [20, 21, 38, 39] yet similar to others [40]. Indeed, a review on adherence in autoimmune conditions also concluded that economic, demographic, and clinical characteristics were only moderately linked to adherence or persistence [24]. The findings on the prospective association of beliefs about medication with adherence and persistence in medication-taking is congruent with previous work in other conditions [41–43]. Still, beliefs about medication were scarcely studied among PwMS; the only study that examined adherence to DMTs was cross-sectional, and found no association between medication beliefs and adherence [16]. Hence, the current study is the first to demonstrate such an association with adherence and persistence. Recent work among PwMS that delved into reasons for non-adherence to DMTs [44] reported avoidance, side effects, cost and mild course of illness; the study reported that the non-adherent group could not be characterized. The present work succeeded in characterizing this group, and suggests that people’s beliefs about medication at onset could predict their adherence and persistence.

The study is hampered by several limitations. First, the study’s sample size is relatively small, and hence the results of the present study should be verified in future studies with a larger cohort of patients. However, this study used medication claims as only one indicator for medication adherence and relied also on patients’ reports of adherence. Secondly, the study was carried out at only one medical center. This may bias the findings, as the patient-practitioner communication [44] and the organizational climate of the specific specialty clinic may not be representative. Thirdly, the study focused on the study period and did not consider the medication history of PwMS (e.g., past pauses or medication switching, [45]). Lastly, the study reported on a one-year follow-up; adherence may still change in a longer follow–up.

The strengths of the study are manifold. First, it relied on multiple measures of adherence: two PROs and medication claims. Second, it measured both adherence and persistence. Third, it included an array of predictors, focusing on patients’ perceptions previously examined only scarcely in adherence to medication among PwMS. The current study addressed four of the five factors in the WHO multidimensional model of medication adherence [46]: social-economic, therapy-related (e.g., type of medication and administration route, naivety), patient-related (e.g., affective states, illness and medication perceptions) and condition-related (e.g., condition duration, EDSS). It did not address health care system characteristics (e.g., monetary issues that could be related to affording a medication), as the study was monocentric, conducted in a socially-financed healthcare system. Lastly, the study used a prospective design that allowed to conclude on prediction.

## Conclusions

To conclude, PwMS’ perceptions of their medication consistently predict adherence and persistence in medication-taking. These findings are similar to conclusions in other medical conditions [47–49]. Other perceptions, such as on one’s health or on one’s illness also predict either adherence or persistence at some time points. Importantly, perceptions are malleable, and can be targeted for potential interventions aimed at increased adherence. Clinicians should therefore discuss with patients their beliefs on their prescribed medication. Lastly, beliefs about medication should be considered as part of a routine PRO battery [15], so as to continually monitor patients’ perceptions about their medication and be able to intervene, if needed. A detailed assessment of beliefs about medication can guide a specific intervention strategy. These are part of implementation of patient empowerment, participatory medicine, and patient-centered approaches [50, 51] in the care of PwMS.

## Data Availability

The datasets analyzed during the current study contain identifying information and is therefore unavailable publicly. Source documents of the research project are securely kept at the MS clinic, Carmel Medical Center, Haifa, Israel. Data can be made available through contacting the last author.

## Abbreviations

B-IPQ: Brief Illness Perception Questionnaire
BMQ: Belief about Medicine Questionnaire
DMTs: Disease-Modifying Therapies
EDSS: Expanded Disability Status Scale
EMD: Electronic Monitoring Device
HADS: Hospital Anxiety and Depression Scale
HMO: Health Maintenance Organization
MPR: Medication Possession Ratio
MRI: Magnetic Resonance Imaging
MS: Multiple Sclerosis
MS-TAQ: Multiple Sclerosis Treatment Adherence Questionnaire
PRO: Patient-Reported Outcomes
ProMAS: Probabilistic Medication Adherence Scale
PwMS: Persons with Multiple Sclerosis
RRMS: Relapsing-Remitting Multiple Sclerosis
SD: Standard Deviation
SRHI: Self Report Habit Index
WHO: World Health Organization
